# A protocol for a mixed-methods analysis of the usability and acceptability of a digital cognitive-behavioural therapy for insomnia (dCBT-I) intervention ( *Sleepio* ^TM^) in participants with cognitive impairment.

**DOI:** 10.12688/hrbopenres.14177.1

**Published:** 2025-06-18

**Authors:** Patrick Crowley, Evelyn Flanagan, Alasdair L. Henry, Rónán O'Caoimh

**Affiliations:** 1 Department of Geriatric Medicine, Mercy University Hospital, Cork, County Cork, T12 WE28, Ireland; 2 Health Research Board Clinical Research Facility-Cork, University College Cork, Mercy University Hospital, Cork, Cork, Ireland, T12 WE28, Ireland; 3 Big Health Limited, London, United Kingdom, UK

**Keywords:** Sleep, Cognitive Impairment, Dementia, Digital Cognitive Behavioural Therapy for Insomnia, Qualitative, Usability, Acceptability.

## Abstract

**Background:**

Sleep disturbance is common among those with cognitive impairment, potentially contributing to negative outcomes. Furthermore, growing evidence suggests that sleep disturbance is associated with the onset and progression of cognitive decline. There is therefore increasing interest in treating sleep disturbance in people with cognitive impairment.

Non-pharmacological treatments for sleep disturbance are unhindered by many of the adverse effects associated with pharmacological treatments, yet evidence supporting their use in clinical practice is often lacking. Cognitive behavioural therapy for insomnia (CBT-I) is the first-line treatment for insomnia and has demonstrated effectiveness in people with cognitive impairment in a small number of trials. Digital CBT-I (dCBT-I) overcomes many of the obstacles associated with accessing CBT-I (such as limited availability of therapists and transportation) whilst presenting others (such as internet access and digital literacy).

**Aim:**

To undertake a mixed-methods analysis of the usability and acceptability of a dCBT-I intervention ( *Sleepio*) among people with cognitive impairment.

**Methods:**

Participants will be community-dwelling adults aged ≥50 years with cognitive decline and probable insomnia. Study partners (being caregivers, family or friends of participants) will be recruited, if available, to examine how their involvement influences usability and acceptability.

Participants will be given access to *Sleepio*, a fully automated and personalised dCBT-I programme delivered over the course of six weekly sessions. Usability and acceptability will be assessed by a bespoke questionnaire incorporating the System Usability Scale and the Usability Metric for User Experience - Lite (UMUX-Lite). Semi-structured interviews will explore participants’ experience of using the intervention.

**Conclusion:**

By examining barriers and facilitators to the usability and acceptability of *Sleepio* among people with cognitive impairment, this study will provide insight into the potential efficacy of the intervention in this population whilst informing the design of a future definitive clinical trial.

## Introduction

Sleep is essential to maintain good physical and mental health ^1– 3^ and plays a central role in many physiological functions ^4^. Nevertheless, insomnia is common with approximately one-third of all adults experiencing insomnia symptoms ^5^, increasing to 75% in those aged over 65 years ^6^. Sleep patterns change as we age ^7^. Normal ageing is associated with reductions in total sleep time, sleep efficiency and slow wave sleep ^8^. Sleep disturbance is even more common among older adults with cognitive impairment ^9^. Compared to healthy aged-matched controls, people with mild cognitive impairment (MCI) experience reduced total sleep time, sleep efficiency and duration of REM, whilst sleep onset latency, wakefulness after sleep onset, and duration of stage one sleep are increased ^10, 11^.

Indeed, there is a well-established association between sleep disturbance and cognitive impairment ^12^, with evidence suggesting that the relationship may be causal and bi-directional ^13^. A meta-analysis of observational studies estimated that 15% of Alzheimer’s Disease may be attributable to sleep disturbance ^14^. Despite the recent Dementia Prevention, Intervention and Care: 2024 report of the Lancet Commission concluding that further research is needed to clarify the effect of sleep disturbance on cognitive decline ^15^, therefore, interest is growing in sleep disturbance as a potential modifiable risk factor for cognitive impairment ^16^. The European Academy of Neurology Brain Health Strategy, for example, has included sleep as a determinant of brain health ^17^.

Furthermore, sleep disturbance tends to worsen as cognitive impairment advances ^18, 19^, leading to deteriorating cognitive outcomes ^20^ and quality of life ^21^, as well as strained relationships with caregivers who experience considerable physical and emotional distress and burnout as a consequence ^22, 23^. The increased caregiver burden caused by sleep disturbance in people with dementia has been shown to lead to sub-optimal care ^24^ and early institutionalisation ^25, 26^. Sleep is therefore an important treatment target throughout the natural history of dementia, to potentially slow the progression of early-stage disease and to reduce the burden of symptoms in more advanced cases.

Pharmacological treatments for sleep disturbance are associated with a range of adverse effects, including sedation and increased risk of falls, headaches and dizziness ^27^. This has led to the American Geriatric Society Beers Criteria recommending against the use of pharmacological agents to treat sleep in older people ^28^. Additionally, there is evidence to suggest that pharmacological treatments to improve sleep can worsen cognitive function in older people ^13^. There is therefore increasing focus on exploring non-pharmacological approaches to treating sleep disturbance among older people with cognitive impairment ^29^.

Cognitive behavioural therapy for insomnia (CBT-I) is the first-line treatment recommended for insomnia and is supported by a large evidence base ^30– 33^. A recent meta-analysis of controlled clinical trials of sleep treatments for people with cognitive impairment included two trials of CBT-I and both demonstrated significant improvements in sleep ^34^. One of the trials, involving 28 participants from independent living facilities, achieved significant improvement in both subjective and objective measures of sleep using the Insomnia Severity Index (ISI) and wrist actigraphy respectively ^35^. The other trial, involving 35 participants recruited from outpatient clinics, found no improvement in objective sleep measures, with significant improvements only in subjective sleep quality ^36^. Participants in the latter trial did not have significant sleep disturbance at baseline, however, and it is perhaps for this reason that meta-analysis of the two trials did not find any significant effect on sleep. Given the promising results in the trial that included participants with sleep disturbances at baseline, there is a need to further investigate the potential application of CBT-I to treat sleep disturbances among people with cognitive impairment.

Unfortunately, however, CBT-I is often difficult to access for many due to the shortage of trained therapists ^37^. Digital CBT-I (dCBT-I) provides a possible solution to overcome the barriers associated with accessing the traditional therapist-delivered model of CBT-I. Indeed, evidence demonstrates the effectiveness of dCBT-I ^38, 39^, which has also recently been shown to be feasible and effective in older people ^40^. A small mixed-methods study involving 12 participants has demonstrated preliminary acceptability of dCBT-I (SHUTi OASIS) among older people with MCI ^41^. More recently, a small open-label randomised controlled feasibility trial demonstrated preliminary efficacy of dCBT-I ( *Sleepio*) in improving the ISI of people with MCI ^42^. This latter trial was conducted remotely, indicating that the participants likely had relatively mild deficits and were adept at using digital technology. A recent qualitative study, conducted among patients who were recovering from stroke, identified some difficulties accessing and operating the *Sleepio* programme ^43^. Nevertheless, nine out of a total of eleven participants successfully completed the programme, although three did require the assistance of their respective caregivers to do so.

It has been suggested that more qualitative research is required to explore barriers and facilitators to the uptake and acceptability of alternative modes of delivery of CBT-I, such as dCBT-I ^44^. By its exploratory and inductive nature ^45^, qualitative research can provide more nuanced and subtle insights into the participants’ perspective of how interventions are delivered in practice ^46^, thus helping to guide the design of future interventions and clinical trials.

## Aim

Given the importance of treating co-morbid sleep disturbance at all stages of cognitive decline, we propose to undertake a mixed-methods analysis of the usability and acceptability of dCBT-I ( *Sleepio*) among people with varying degrees of cognitive decline, ranging from subjective cognitive decline (SCD) through to MCI and mild-moderate dementia. We will also endeavour to assess whether usability and acceptability can be improved by the availability of a study partner to assist with operation of the dCBT-I ( *Sleepio*) programme. This will help to determine which people with cognitive impairment may be able to use and benefit from dCBT-I ( *Sleepio*) to treat co-morbid sleep disturbance.

## Methods

### Intervention

*Sleepio* is a fully-automated and personalised dCBT-I intervention designed to treat insomnia using a combination of cognitive, behavioural, sleep hygiene and relaxation techniques. It is delivered through a web-based platform, supporting iOS or Android mobile applications. Underlying algorithms organise the delivery of information, support, and advice in a manner tailored to each individual patient based on their responses to an initial sleep study questionnaire and subsequent daily sleep diary entries throughout the duration of the programme. The programme consists of six sessions, each between five and twenty minutes in duration. *Sleepio* can be completed within a six-week period. However, the average time taken to complete the programme is 9–10 weeks. No time restriction is placed on the user to complete the sessions, and all previously viewed sessions remain available to the user to re-watch throughout the duration of the programme. Participants are offered daily notifications to prompt adherence to the programme.

### Participants

Potential participants will be community-dwelling adults aged fifty years and older with probable insomnia attending outpatient memory clinics affiliated with the Mercy University Hospital in Cork, Ireland. Only participants with probable insomnia will be included because, although previous studies have suggested the effectiveness of *Sleepio* in sub-clinical insomnia ^47^, other studies have demonstrated that participants’ motivation to adhere to the *Sleepio* programme was proportional to the severity of their sleep disturbance ^43^. Study partners, if available, will also be recruited for dyadic participation and may include caregivers, family members or friends attending memory clinic with the participants. A previous qualitative study of the usability of *Sleepio* amongst patients following stroke found that some had to rely on help from partners to operate the programme and suggested that more people may be able to use dCBT-I if given support from a partner or caregiver ^43^. It is anticipated that study partners may play an important role in facilitating successful adherence of participants to the *Sleepio* programme, particularly among participants with more advanced cognitive impairment ^48^. While ideal and likely to enrich the study, the availability of a study partner will not be an inclusion/exclusion criterion. We will nevertheless endeavour to include them when available. Other inclusion/exclusion criteria are as follows:

Inclusion criteria:

1. Aged ≥50 years.
2. Sleep Condition Indicator (SCI) ^49^ Score ≤16/32. The SCI is an 8 item scale validated to assess insomnia based on DSM-5 criteria. Scores range from 0–32, with scores ≤16 indicating probable insomnia.
3. Established SCD, MCI or mild-moderate dementia, diagnosed by a consultant physician specialised in cognitive disorders prior to enrolment in the study. Dementia will be defined using DSM-5 classification and staged according to the Reisberg FAST scale ^50^ into early/mild/moderate stage. MCI will be defined using Petersen’s criteria ^51^. Participants must score between 0 – 2 on the Clinical Dementia Rating (CDR) scale ^52^, a commonly used clinician-rating scale of global symptom severity in dementia.
4. Sufficient physical/sensory (visual/hearing) capacity to use the intervention, as judged by the clinician/investigator.
5. English-speaker (intervention only available in English).
6. Community-dwellers (non-institutionalised).
7. Ability to provide informed written consent.

Exclusion criteria:

1. Known history of an International Classification of Diseases (ICD) defined sleep disorder other than insomnia disorder.
2. Receipt of a sleep-related cognitive behavioural therapy intervention within the past six months.
3. CDR >2
4. Severe depression (defined as depression requiring hospitalization in the past 12-months or visit to psychiatry outpatient clinic in the past three months).
5. Unstable depression/anxiety disorders or panic attacks (unstable will be defined as changes in antidepressant medications within the last three months - i.e. no start, stop or change in dose).
6. Other relevant major neuropsychiatric disorders, including schizophrenia, psychosis, mania, bipolar affective disorder, epilepsy or seizure disorder.
7. Ongoing substance or alcohol abuse.
8. Long-term physical or sensory impairment, pain or other medical condition which, in the opinion of the principal investigator, could impair participation for reasons other than cognitive impairment.
9. Planned surgery or hospitalisation during the study that could interfere with participation.
10. Medical conditions rendering the patient too unwell to continue to participate in the study in the opinion of the principal investigator.
11. A change in the following medication within the three months prior to enrolment:
  a. Benzodiazepine and non-benzodiazepine hypnotic or anxiolytics agents
  b. Cholinesterase inhibitors or Memantine
  c. Antidepressants
  d. Antipsychotics
  e. Amphetamine derivatives
  f. Decongestants
  g. Narcotic analgesics
  h. β-Blockers
  i. Pulmonary medications (Theophylline and Albuterol)
  j. Melatonin

### Recruitment

All patients attending memory clinics affiliated with the Mercy University Hospital in Cork, Ireland will be screened for eligibility. Consecutive patients who meet eligibility criteria will be invited to participate in the study. It is hoped to recruit 20–30 participants over a four month period. Where possible, study partners including friends or family members of participants, will be recruited to support participants throughout the study.

### Consent process

Fully informed written consent will be obtained from each participant prior to enrolment. The capacity of all participants to provide valid informed consent to participate in the study will be determined by a consultant physician in geriatric medicine. Fully informed written consent to participation will also be obtained from study partners (caregivers, friends, and or family members) if available and willing to participate. Participants and their study partners (if applicable) will be provided with a Participant Information Leaflet detailing the purpose of the study and what participation in the study would entail for them. Copies of the Participant Information Leaflet and Consent Forms that will be used in the study are available in the extended data.

### Enrolment

Once recruited, participants will be provided with access to the study intervention ( *Sleepio*). Given the digital nature of the intervention, each participant’s digital literacy will be assessed using the Mobile Device Proficiency Questionnaire ^53^. Every participant will be provided with a training/orientation session to teach them how to use the *Sleepio* programme. Additionally, each participant will be provided with clear written instructions on how to access and use the *Sleepio* programme. If a study partner is available, and both the participant and the study partner consent to their participation in the study, the partner will be also included in the training session.

### Data collection

Data collection will be carried out in accordance with the European Union’s General Data Protection Regulation and local data protection policies. The following data will be recorded in respect of every participant at baseline:

- Demographic data, including: age, sex, marital status, level of education, and employment status.
- Charlson Co-morbidity Index ^54^
- Cognitive diagnosis and stage of cognitive impairment
- Medications

Following completion of the *Sleepio* programme, usability and acceptability will be assessed initially by a bespoke questionnaire incorporating the System Usability Scale ^55^, adapted for cognitive impairment ^56^, and the Usability Metric for User Experience - Lite (UMUX -Lite) ^57^. The System Usability Scale is a validated and commonly employed 10 item Likert-type scale that provides a global assessment of how user-friendly a system or product is perceived to be. The UMUX-Lite is a more condensed two item Likert-type scale that also measures user-satisfaction with a system or product. Finally, participants and their study partners (where applicable) will be interviewed to further assess the usability and acceptability of the programme. The interview will be audio-recorded and conducted in a semi-structured manner using the topic guide included in the supplementary material. Participants and their study partners will be asked to expound on any difficulties they had in complying with the programme and whether they found the programme beneficial. In order to reduce the possibility of social desirability and positivity bias, the participants and their study partners (where applicable) will be informed that the interviewer is not affiliated with *Sleepio* and that the objective of the study is to obtain an accurate understanding of their experience using the programme in order to guide future research.

### Data analysis

In respect of the bespoke questionnaire on usability and acceptability, categorical variables will be described in frequencies and percentages, while mean scores will be reported for the incorporated Systems Usability Scale and UMUX-Lite. 

The audio-recorded semi-structured interviews will be transcribed verbatim. Transcripts of the interviews will be anonymised and the audio-recordings will be destroyed immediately following transcription. Transcripts will be read and re-read to ensure data immersion. Thematic analysis will be undertaken using open coding to identify patterns and themes in a six-stage process involving: familiarisation, generating initial codes, developing themes, reviewing themes, defining and naming themes, and reporting findings ^46^. This process of analysis will be undertaken manually at first and then repeated using a qualitative data management software system to ensure thorough analysis. Emerging patterns and themes will be discussed periodically amongst all the researchers involved in this study (except ALH – see competing interests statement below) to facilitate collaborative reflexive analysis as consensus is reached ^58^. The Standards for Reporting Qualitative Research will be adhered to when reporting the findings of the study ^59^.

### Discussion

This mixed-methods study will explore the usability and acceptability of a dCBT-I ( *Sleepio*) intervention in people with cognitive impairment. CBT-I is the first-line treatment for insomnia ^30– 33^. However, the effectiveness of cognitive behavioural therapy may be limited in people with cognitive impairment by their diminished ability to comprehend, learn, remember and apply the skills taught by the therapy programme ^48^. This may be more marked for dCBT-I. By including participants with cognitive impairment ranging from SCD to mild-moderate dementia, this study will help identify the categories of people from amongst this cohort who are most likely to benefit from dCBT-I. By examining the barriers and facilitators that influence usability and acceptability of the *Sleepio* programme amongst people with cognitive impairment, this study will investigate how the delivery of dCBT-I may be adapted to enhance usability and acceptability amongst this population. In particular, the effect of digital literacy and the availability of study partners to support adherence to the programme will be analysed. Improved understanding of the influence of these factors will assist in the development of guidance on the use and application of dCBT-I in clinical practice for this cohort and older adults in general, who often rate their own comfort and familiarity with technology as poor or average ^60^. By providing insights into the factors that influence usability, acceptability and potential efficacy of dCBT-I amongst people with cognitive impairment, this study will also help inform the design of a future definitive randomised controlled trial that will assess the effectiveness of dCBT-I to treat probable insomnia amongst this population.

## Conclusion

This mixed-methods analysis of the usability and acceptability of a dCBT-I ( *Sleepio*) intervention in people with cognitive impairment will help inform the design of a future randomised controlled trial that will assess the effectiveness of the intervention in this population.

## Ethics & consent statement

This mixed-methods qualitative study will be conducted in accordance with the Declaration of Helsinki. Ethical approval for this study was granted by the Clinical Research Ethics Committee of the Cork Teaching Hospitals (CREC) on the 5 ^th^ December 2023, under CREC reference number: ECM 4 (gg) 05/12/2023. Results of the study will be published in a peer-reviewed journal and presented at relevant academic conferences. Fully informed written consent will be obtained from each participant prior to enrolment.

## Data Availability

No data are associated with this article. Open Science Framework: A protocol for a mixed-methods analysis of the usability and acceptability of a digital cognitive-behavioural therapy for insomnia (dCBT-I) intervention ( *Sleepio*) in participants with cognitive impairment. https://doi.org/10.17605/OSF.IO/U8CBY This article contains the following extended data ^61^: Interview Topic Guide Participant Information Leaflet & Consent Form (Participant) Participant Information Leaflet & Consent Form (Study Partner) Open Science Framework: A protocol for a mixed-methods analysis of the usability and acceptability of a digital cognitive-behavioural therapy for insomnia (dCBT-I) intervention ( *Sleepio*) in participants with cognitive impairment. https://doi.org/10.17605/OSF.IO/U8CBY This article contains the following reporting guidelines ^61^: Standards for Reporting Qualitative Research Data are available under the terms of the Creative Commons Zero "No rights reserved" data waiver (CC0 1.0 universal).

## References

[1] Kyle SD Henry AL : Sleep is a modifiable determinant of health: implications and opportunities for health psychology. *Br J Health Psychol.* 2017;22(4):661–670. 10.1111/bjhp.12251 28972306

[2] Lim DC Najafi A Afifi L : The need to promote sleep health in public health agendas across the globe. *Lancet Public Health.* 2023;8(10):e820–e826. 10.1016/S2468-2667(23)00182-2 37777291 PMC10664020

[3] Luyster FS Strollo PJ Jr Zee PC : Sleep: a health imperative. *Sleep.* 2012;35(6):727–34. 10.5665/sleep.1846 22654183 PMC3353049

[4] Walker M : Why we sleep.Harlow, England: Penguin Books,2018. Reference Source

[5] Krueger PM Friedman EM : Sleep duration in the United States: a cross-sectional population-based study. *Am J Epidemiol.* 2009;169(9):1052–63. 10.1093/aje/kwp023 19299406 PMC2727237

[6] Nguyen V George T Brewster GS : Insomnia in older adults. *Curr Geriatr Rep.* 2019;8(4):271–290. 33312842 10.1007/s13670-019-00300-x PMC7731454

[7] Bliwise DL : Sleep in normal aging and dementia. *Sleep.* 1993;16(1):40–81. 10.1093/sleep/16.1.40 8456235

[8] Ohayon MM Carskadon MA Guilleminault C : Meta-analysis of quantitative sleep parameters from childhood to old age in healthy individuals: developing normative sleep values across the human lifespan. *Sleep.* 2004;27(7):1255–73. 10.1093/sleep/27.7.1255 15586779

[9] Guarnieri B Adorni F Musicco M : Prevalence of sleep disturbances in mild cognitive impairment and dementing disorders: a multicenter Italian clinical cross-sectional study on 431 patients. *Dement Geriatr Cogn Disord.* 2012;33(1):50–8. 10.1159/000335363 22415141 PMC3696366

[10] D'Rozario AL Chapman JL Phillips CL : Objective measurement of sleep in mild cognitive impairment: a systematic review and meta-analysis. *Sleep Med Rev.* 2020;52: 101308. 10.1016/j.smrv.2020.101308 32302775

[11] Wei J Wang M Guo Y : Sleep structure assessed by objective measurement in patients with mild cognitive impairment: a meta-analysis. *Sleep Med.* 2024;113:397–405. 10.1016/j.sleep.2023.12.010 38134714

[12] Shi L Chen SJ Ma MY : Sleep disturbances increase the risk of dementia: a systematic review and meta-analysis. *Sleep Med Rev.* 2018;40:4–16. 10.1016/j.smrv.2017.06.010 28890168

[13] Mander BA Winer JR Jagust WJ : Sleep: a novel mechanistic pathway, biomarker, and treatment target in the pathology of Alzheimer's disease? *Trends Neurosci.* 2016;39(8):552–566. 10.1016/j.tins.2016.05.002 27325209 PMC4967375

[14] Bubu OM Brannick M Mortimer J : Sleep, cognitive impairment, and Alzheimer's disease: a systematic review and meta-analysis. *Sleep.* 2017;40(1). 28364458 10.1093/sleep/zsw032

[15] Livingston G Huntley J Liu KY : Dementia prevention, intervention, and care: 2024 report of the *Lancet* standing Commission. *Lancet.* 2024;404(10452):572–628. 10.1016/S0140-6736(24)01296-0 39096926

[16] Minakawa EN Wada K Nagai Y : Sleep disturbance as a potential modifiable risk factor for Alzheimer's disease. *Int J Mol Sci.* 2019;20(4):803. 10.3390/ijms20040803 30781802 PMC6412395

[17] Bassetti CLA Endres M Sander A : The European Academy of Neurology Brain Health Strategy: one brain, one life, one approach. *Eur J Neurol.* 2022;29(9):2559–2566. 10.1111/ene.15391 35538709

[18] Liguori C Placidi F Izzi F : Sleep dysregulation, memory impairment, and CSF biomarkers during different levels of neurocognitive functioning in Alzheimer's disease course. *Alzheimers Res Ther.* 2020;12(1): 5. 10.1186/s13195-019-0571-3 31901236 PMC6942389

[19] Prinz PN Vitaliano PP Vitiello MV : Sleep, EEG and mental function changes in senile dementia of the Alzheimer's type. *Neurobiol Aging.* 1982;3(4):361–70. 10.1016/0197-4580(82)90024-0 7170052

[20] Wennberg AMV Wu MN Rosenberg PB : Sleep disturbance, cognitive decline, and dementia: a review. *Semin Neurol.* 2017;37(4):395–406. 10.1055/s-0037-1604351 28837986 PMC5910033

[21] Vitiello MV Borson S : Sleep disturbances in patients with Alzheimer's disease: epidemiology, pathophysiology and treatment. *CNS Drugs.* 2001;15(10):777–96. 10.2165/00023210-200115100-00004 11602004

[22] Donaldson C Tarrier N Burns A : Determinants of carer stress in Alzheimer's disease. *Int J Geriatr Psychiatry.* 1998;13(4):248–256. 10.1002/(sici)1099-1166(199804)13:4<248::aid-gps770>3.0.co;2-0 9646153

[23] Pudelewicz A Talarska D Bączyk G : Burden of caregivers of patients with Alzheimer's disease. *Scand J Caring Sci.* 2019;33(2):336–341. 10.1111/scs.12626 30378698

[24] McCurry SM Vitiello MV Gibbons LE : Factors associated with caregiver reports of sleep disturbances in persons with dementia. *Am J Geriatr Psychiatry.* 2006;14(2):112–20. 10.1097/01.JGP.0000192499.25940.da 16473975

[25] Gaugler JE Edwards AB Femia EE : Predictors of institutionalization of cognitively impaired elders: family help and the timing of placement. *J Gerontol B Psychol Sci Soc Sci.* 2000;55(4):P247–P255. 10.1093/geronb/55.4.p247 11584881

[26] Pollak CP Perlick D Linsner LP : Sleep problems in the community elderly as predictors of death and nursing home placement. *J Community Health.* 1990;15(2):123–35. 10.1007/BF01321316 2355110

[27] Lam S Macina LO : Therapy update for Insomnia in the elderly. *Consult Pharm.* 2017;32(10):610–622. 10.4140/TCP.n.2017.610 28992822

[28] By the 2023 American Geriatrics Society Beers Criteria® Update Expert Panel: American Geriatrics Society 2023 updated AGS Beers Criteria ^®^ for potentially inappropriate medication use in older adults. *J Am Geriatr Soc.* 2023;71(7):2052–2081. 10.1111/jgs.18372 37139824 PMC12478568

[29] O'Caoimh R Mannion H Sezgin D : Non-pharmacological treatments for sleep disturbance in mild cognitive impairment and dementia: a systematic review and meta-analysis. *Maturitas.* 2019;127:82–94. 10.1016/j.maturitas.2019.06.007 31351523

[30] Edinger JD Arnedt JT Bertisch SM : Behavioral and psychological treatments for chronic insomnia disorder in adults: an American Academy of Sleep Medicine clinical practice guideline. *J Clin Sleep Med.* 2021;17(2):255–262. 10.5664/jcsm.8986 33164742 PMC7853203

[31] Qaseem A Kansagara D Forciea MA : Management of chronic insomnia disorder in adults: a clinical practice guideline from the American College of Physicians. *Ann Intern Med.* 2016;165(2):125–33. 10.7326/M15-2175 27136449

[32] Riemann D Espie CA Altena E : The European Insomnia guideline: an update on the diagnosis and treatment of insomnia 2023. *J Sleep Res.* 2023;32(6): e14035. 10.1111/jsr.14035 38016484

[33] Wilson S Anderson K Baldwin D : British Association for Psychopharmacology consensus statement on evidence-based treatment of insomnia, parasomnias and circadian rhythm disorders: an update. *J Psychopharmacol.* 2019;33(8):923–947. 10.1177/0269881119855343 31271339

[34] Crowley P Flanagan E O'Caoimh R : A protocol for the systematic review and meta-analysis of clinical trials of interventions to improve sleep in people with mild cognitive impairment or dementia. [version 1; peer review: 1 approved with reservations]. *HRB Open Res.* 2024;7:63. 10.12688/hrbopenres.13988.1

[35] Cassidy-Eagle E Siebern A Unti L : Neuropsychological functioning in older adults with mild cognitive impairment and Insomnia randomized to CBT-I or control group. *Clin Gerontol.* 2018;41(2):136–144. 10.1080/07317115.2017.1384777 29220627

[36] Naismith SL Pye J Terpening Z : "Sleep Well, Think Well" group program for mild cognitive impairment: a randomized controlled pilot study. *Behav Sleep Med.* 2019;17(6):778–789. 10.1080/15402002.2018.1518223 30247939

[37] Koffel E Kuhn E Petsoulis N : A randomized controlled pilot study of CBT-I Coach: Feasibility, acceptability, and potential impact of a mobile phone application for patients in cognitive behavioral therapy for insomnia. *Health Informatics J.* 2018;24(1):3–13. 10.1177/1460458216656472 27354394

[38] Soh HL Ho RC Ho CS : Efficacy of digital cognitive behavioural therapy for insomnia: a meta-analysis of randomised controlled trials. *Sleep Med.* 2020;75:315–325. 10.1016/j.sleep.2020.08.020 32950013

[39] Zachariae R Lyby MS Ritterband LM : Efficacy of internet-delivered cognitive-behavioral therapy for insomnia – a systematic review and meta-analysis of randomized controlled trials. *Sleep Med Rev.* 2016;30:1–10. 10.1016/j.smrv.2015.10.004 26615572

[40] Lee Y Kim I Lee S : Information and communication technology-based application for cognitive behavioral therapy among community-dwelling older adults with Insomnia: development and validation study. *Healthcare (Basel).* 2024;12(1):106. 10.3390/healthcare12010106 38201011 PMC10778576

[41] Mattos MK Manning CA Quigg M : Feasibility and preliminary efficacy of an Internet-delivered intervention for insomnia in individuals with Mild Cognitive Impairment. *J Alzheimers Dis.* 2021;84(4):1539–1550. 10.3233/JAD-210657 34690141 PMC10445244

[42] Hoyos C Espinosa N Marshall N : 0441 sleep disturbance in MCI: a pilot study of a Cognitive Behavioural Therapy digital intervention (SUCCEED). *Sleep.* 2024;47(Suppl 1):A189. 10.1093/sleep/zsae067.0441

[43] Smejka T Henry AL Wheatley C : A qualitative examination of the usability of a digital Cognitive Behavioral Therapy for Insomnia program after stroke. *Brain Inj.* 2022;36(2):271–278. 10.1080/02699052.2022.2034182 35108134 PMC9162493

[44] Koffel E Bramoweth AD Ulmer CS : Increasing access to and utilization of Cognitive Behavioral Therapy for Insomnia (CBT-I): a narrative review. *J Gen Intern Med.* 2018;33(6):955–962. 10.1007/s11606-018-4390-1 29619651 PMC5975165

[45] Savin-Baden M Major CH : Qualitative research: the essential guide to theory and practice.Routledge,2023. 10.4324/9781003377986

[46] Braun V Clarke V : Using thematic analysis in psychology. *Qual Res Psychol.* 2006;3(2):77–101. 10.1191/1478088706qp063oa

[47] Denis D Eley TC Rijsdijk F : Is digital Cognitive Behavioural Therapy for Insomnia effective in treating sub-threshold insomnia: a pilot RCT. *Sleep Med.* 2020;66:174–183. 10.1016/j.sleep.2019.10.007 31901759

[48] Kraus CA Seignourel P Balasubramanyam V : Cognitive-Behavioral Treatment for anxiety in patients with dementia: two case studies. *J Psychiatr Pract.* 2008;14(3):186–92. 10.1097/01.pra.0000320120.68928.e5 18520790 PMC2567867

[49] Espie CA Kyle SD Hames P : The Sleep Condition Indicator: a clinical screening tool to evaluate insomnia disorder. *BMJ Open.* 2014;4(3): e004183. 10.1136/bmjopen-2013-004183 24643168 PMC3964344

[50] Reisberg B : Functional Assessment Staging (FAST). *Psychopharmacol Bull.* 1988;24(4):653–659. 3249767

[51] Petersen RC Smith GE Waring SC : Mild cognitive impairment: clinical characterization and outcome. *Arch Neurol.* 1999;56(3):303–8. 10.1001/archneur.56.3.303 10190820

[52] Morris JC : The Clinical Dementia Rating (CDR): current version and scoring rules. *Neurology.* 1993;43(11):2412–4. 10.1212/wnl.43.11.2412-a 8232972

[53] Roque NA Boot WR : A new tool for assessing mobile device proficiency in older adults: the Mobile Device Proficiency Questionnaire. *J Appl Gerontol.* 2018;37(2):131–156. 10.1177/0733464816642582 27255686 PMC9394541

[54] Charlson ME Pompei P Ales KL : A new method of classifying prognostic comorbidity in longitudinal studies: development and validation. *J Chronic Dis.* 1987;40(5):373–83. 10.1016/0021-9681(87)90171-8 3558716

[55] Lewis JR : The System Usability Scale: past, present, and future. *Int J Hum Comput Interact.* 2018;34(7):577–590. 10.1080/10447318.2018.1455307

[56] Holden RJ : A simplified System Usability Scale (SUS) for cognitively impaired and older adults. *Proc Int Symp Hum Factors Ergon Healthc.* 2020;9(1):180–182. 10.1177/2327857920091021

[57] Lewis JR Utesch BS Maher DE : UMUX-LITE: when there's no time for the SUS.In: *Proceedings of the SIGCHI Conference on Human Factors in Computing Systems.*Association for Computing Machinery: Paris, France,2013;2099–2102. 10.1145/2470654.2481287

[58] Olmos-Vega FM Stalmeijer RE Varpio L : A practical guide to reflexivity in qualitative research: AMEE guide no. 149. *Med Teach.* 2023;45(3):241–251. 10.1080/0142159X.2022.2057287 35389310

[59] O’Brien BC Harris IB Beckman TJ : Standards for Reporting Qualitative Research: a synthesis of recommendations. *Acad Med.* 2014;89(9):1245–1251. 10.1097/ACM.0000000000000388 24979285

[60] Scanlon L O'Shea E O'Caoimh R : Technology use and frequency and self-rated skills: a survey of community-dwelling older adults. *J Am Geriatr Soc.* 2015;63(7):1483–4. 10.1111/jgs.13507 26189859

[61] Crowley P Flanagan E O’Caoimh R : A protocol for a mixed-methods analysis of the usability and acceptability of a digital Cognitive-Behavioural Therapy for Insomnia (dCBT-I) intervention (Sleepio) in participants with cognitive impairment. 10.17605/OSF.IO/U8CBY PMC12828257 41584366

