# Supplementary material for: A protocol for a mixed-methods analysis of the usability and acceptability of a digital cognitive-behavioural therapy for insomnia (dCBT-I) intervention ( Sleepio TM) in participants with cognitive impairment
Source: HRB Open Res. 2025 Jun 18;8:68. [Version 1] doi: 10.12688/hrbopenres.14177.1 (PMC12828257; doi:10.12688/hrbopenres.14177.1)
Supplement: Peer review history

# Reviewer report: Reviewer response for version 1

**DOI:** 10.21956/hrbopenres.15590.r52555

**Published:** 2026-01-22

**Authors:** Inga Antonsdottir

**Affiliations:** 1 Johns Hopkins Medicine, Baltimore, Maryland, USA

Review for: A protocol for a mixed-methods analysis of the usability and acceptability of a digital cognitive-behavioural therapy for insomnia (dCBT-I) intervention ( *Sleepio* ^TM^) in participants with cognitive impairment.

 General comment: Thank you for the opportunity to review this well-written and relevant manuscript. The manuscript outlines a protocol for the important topic of understanding the usability and acceptance of a digital cognitive behavioral therapy for insomnia intervention for people with cognitive impairment.

 Abstract: Clear and well-written.

 Keywords: appropriate.

 Introduction:  

- This sentence in the second paragraph, *“Despite the recent Dementia Prevention, Intervention and Care: 2024 report of the Lancet Commission concluding that further research is needed to clarify the effect of sleep disturbance on cognitive decline ^15^, therefore, interest is growing in sleep disturbance as a potential modifiable risk factor for cognitive impairment ^16^.” *Is a little long-winded and difficult to follow. Please consider breaking it up.

Methods:

- There is no time restriction placed on the user to complete the sessions, it may be beneficial to place a generous time restriction to allow participants some sort of guideline to follow, such as 12 or 15 weeks? Or does time restriction refer to the number of hours it takes for participants to finish a session? Please clarify – thanks!
- Anxiety and depression disorders are listed, are there any neuropsychiatric symptoms that could impact the intervention that you may want to exclude? Such as nighttime agitation, sundowning, wandering? Potentially add to exclusion number 6, “other relevant major NEUROPSYCHIATRIC SYMPTOMS or major neuropsychiatric disorders…”?
- In the recruitment section, consider adding how many patients are seen in the clinic during a month.

Discussion & Conclusion: well written, no comments.

Is the study design appropriate for the research question?

Yes

Is the rationale for, and objectives of, the study clearly described?

Yes

Are sufficient details of the methods provided to allow replication by others?

Yes

Are the datasets clearly presented in a useable and accessible format?

Yes

Reviewer Expertise:

dementia, caregiving, sleep, cbti

I confirm that I have read this submission and believe that I have an appropriate level of expertise to confirm that it is of an acceptable scientific standard.

# Reviewer report: Reviewer response for version 1

**DOI:** 10.21956/hrbopenres.15590.r48112

**Published:** 2025-08-18

**Authors:** Alexandra Hinterberger

**Affiliations:** 1 Salzburg University, Salzburg, Austria

The authors propose a mixed-methods protocol to investigate the usability and acceptability of a dCBT-I program (Sleepio) in participants with cognitive impairment. 20-30 adults aged 50+ with MCI and likely insomnia will use Sleepio over (a minimum of) six weeks. Questionnaires and interviews will evaluate the program’s usability and user experience. Study partners (caregivers or relatives) will also be included to assess their influence.

 Overall, the authors present a well thought-through study design suitable for addressing the main question of interest and include various adequate measures.

 Including only patients with probable insomnia is obvious to investigate the usability of a dCBT-I program in an affected population. 

 I particularly endorse the use of not only questionnaires, but also semi-structured interviews for the assessment of participants' program experience to get a more realistic and profound perspective. It is however, to be accounted for answers biased by social desirability (even when explicitly mentioning that the interviewer is not affiliated with Sleepio). Furthermore, I appreciate the assessment of participants' digital literacy to account for potential difficulties due to the digital nature of the CBT-I program.

 The interview questions provided seem to cover the most relevant aspects while leaving room for open answers and conversation. 

 While the protocol covers the most important points, some questions/points require further consideration:

 1) How will the authors ensure, that participants with cognitive impairment will fully understand the questionnaire items to assess usability? Will the questionnaires be read and explained to participants if needed?

 2) How will the progress of the dCBT-I program be tracked (completion of exercises, duration of time spent in the program, etc)? And how does this align with data protection regulations? 

 3) Did the authors plan on including any forms of objective sleep measurement (PSG, actigraphy, HRV-based measurements)? Or will the effects of the dCBT-I program on sleep be solely assessed using the subjective experience?

 4) Is there a specific reason as to why the Insomnia Severity Index (ISI) was not included in this protocol? Including it would foster comparability with other, similar study protocols as described by the authors in the introduction. 

 5) The dCBT-I program is being tailored to the individual. To what extend does the content of the program vary (e.g., are all patients still presented with all core elements of CBT-I, like bed time restriction)? And if the content delivered differs significantly between patients, how do the authors ensure comparability of the usability and acceptability? 

 6) Speaking of bed time restriction: How will the adherence to more challenging elements of dCBT-I be ensured/monitored? Or are participants free to choose what exercises they follow through?

 7) Please provide a short overview regarding the questions participants will have to answer (daily?) in the sleep diary. 

 8) The authors say that no time restriction will be placed regarding the completion of the program, Does this mean that study participants will have up to 4 months (duration of the study) to complete the 6 sessions? If so, is the progress between participants and therefore potential stress and beneficial effects still comparable? 

 9) The authors state "Receipt of sleep-related cognitive behavioral therapy intervention within the past six months" as an exclusion criterion. I am wondering whether six months might be too little time to show (potential) effects of the dCBT-I program. The authors might benefit from either excluding patients who have already completed CBT-I, or increasing the time that should have passed between the interventions. 

 10) In the Participant Information Document, please add that the interview will be recorded for full transparency.

Is the study design appropriate for the research question?

Yes

Is the rationale for, and objectives of, the study clearly described?

Yes

Are sufficient details of the methods provided to allow replication by others?

Partly

Are the datasets clearly presented in a useable and accessible format?

Not applicable

Reviewer Expertise:

psychology, sleep research, insomnia, CBT-I

I confirm that I have read this submission and believe that I have an appropriate level of expertise to confirm that it is of an acceptable scientific standard.
